# Anxiety and Depression after Myocardial Infarction: Can Inflammatory Factors be Involved?

**DOI:** 10.5935/abc.20180233

**Published:** 2018-11

**Authors:** João Manoel Theotonio dos Santos

**Affiliations:** Escola de Ciências da Saúde - Curso de Medicina - Universidade Anhembi Morumbi, São José dos Campos, SP - Brazil

**Keywords:** Myocardial Infarction, Anxiety, Depression, Risk Factors, Inflammation, Gender Identity, C-Reactive Protein

This interesting article published by Serpytis et al.,^1^ evaluated the presence of depression and anxiety disorders after acute myocardial infarction, and the different forms of presentation and prevalence according to patient gender and age.

The authors observed that over a period of up to 31 days after an acute myocardial infarction, more than two-thirds of the patients had depression and /or anxiety disorders. Women had a higher prevalence of these comorbidities when compared to men and also tended to have more severe presentations of both depression and anxiety disorders. Additionally, in men, depression was more severe and anxiety disorder was less severe as they were older; whereas in women these comorbidities showed a linear presentation regarding severity, regardless of the age factor.^1^

Other interesting points were that diabetic and / or sedentary men showed a higher score of depression, whereas men who smoked had a higher anxiety score. Regarding hypercholesterolemia, it was observed that women showed higher scores for depression and anxiety disorder, which did not occur with men.

Also, regarding risk factors for coronary artery disease, a sedentary lifestyle was associated with higher scores of depression and anxiety disorder in women.

Finally, it is noteworthy the fact that systemic arterial hypertension and body mass index were not associated at all to the presence of depression and/or anxiety disorder. Considering the data presented herein, despite the limitations already described by the authors, one can say there is a high prevalence of depression and anxiety disorder in the 31 days following acute myocardial infarction.^1^

Literature data show us that the association of some risk factors for coronary artery disease, such as diabetes mellitus, hypercholesterolemia, smoking and a sedentary lifestyle, has been studied in the last two decades and the studies agree regarding their association with depression and anxiety disorder in these patients.^2^^-^^6^

As for the mechanism that could trigger depression and anxiety disorder after acute myocardial infarction, it might be explained as a type of post-traumatic stress, in which individuals affected by a disease that puts them at risk of impending death makes them think about how their life will be altered after this clinical event, such as changes in habits, possible sequelae, and limitations to the activities of daily living. The disease experience can precipitate stressful feelings and reactions, which include pictures of depression and anxiety disorder.^7^^,^^8^

Moreover, in recent years, when searching for new concepts to understand the development of depression, and so come up with better treatments, research has demonstrated the immune system participation, particularly the inflammatory response, as a potentially important contributor to the pathophysiology of depression.^9^ It is noteworthy the fact that these inflammatory factors, such as C-reactive protein, TNF-α and Interleukin-6 are also elevated in the acute phase of myocardial infarction.^10^

Finally, it is very interesting that two diseases with a strong association with inflammatory factors appear concomitantly and with their prevalence presented herein.

We hope future studies will be designed with the specific aim of elucidating this interesting association.
